# Editorial: Cell therapy in the central nervous system and its crosstalk with the immune system

**DOI:** 10.3389/fncel.2026.1916972

**Published:** 2026-07-09

**Authors:** María Norte-Munoz, Raquel Boia, David García-Bernal, Marta Agudo-Barriuso

**Affiliations:** 1 Experimental Ophthalmology Research Group, Department of Ophthalmology, Optometry, Otorhinolaryngology, and Pathological Anatomy, Faculty of Optics and Optometry, Instituto Murciano de Investigación Biosanitaria Pascual Parrilla (IMIB), University of Murcia, Murcia, Spain; 2 Coimbra Institute for Clinical and Biomedical Research (iCBR), Faculty of Medicine, University of Coimbra, Coimbra, Portugal; 3 Hematopoietic Transplantation and Cellular Therapy Research Group, Department of Biochemistry, Molecular Biology and Immunology, Faculty of Medicine, Instituto Murciano de Investigación Biosanitaria Pascual Parrilla (IMIB), University of Murcia, Murcia, Spain

**Keywords:** cell therapy, central nervous system, extracellular vesicles (EVs), multiple sclerosis, regeneration, stroke

The central nervous system (CNS) has long been considered an immune-privileged site. However, this prevailing view is changing. Current evidence shows that immune responses are central to neural homeostasis, degeneration, and repair. Indeed, neuroinflammation is now recognized as a common denominator across a broad spectrum of neurological disorders, including autoimmune, neurodegenerative, vascular, and traumatic diseases. In this context, the Research Topic *Cell therapy in the central nervous system and its crosstalk with the immune system* brings together studies that examine how cell-based therapies interact with neuroimmune pathways across the brain, spinal cord, and retina. The goal is to move beyond simple repair and understand how immune recognition, inflammation, and glial activity shape therapeutic efficacy and clinical translation. Collectively, these contributions reflect an emerging paradigm in which successful regenerative therapies are those capable of simultaneously promoting tissue repair and modulating dysregulated immune responses.

This perspective is illustrated in *The evolution of mesenchymal stem cell-derived neural progenitor therapy for Multiple Sclerosis: from concept to clinic*. Current treatments for multiple sclerosis (MS) mainly target immune responses but have limited effects on neurodegeneration. This review presents mesenchymal stem cell-derived neural progenitors (MSC-NPs) as a dual strategy that combines immunomodulation with repair. It traces their development from preclinical studies to early clinical use, while highlighting the need to better define mechanisms of action and confirm long-term safety and efficacy. Importantly, MSC-NPs exemplify how cell-based interventions can influence both arms of disease pathology by suppressing pathogenic immune activity while promoting endogenous regenerative processes, including remyelination and neuroprotection.

Extending this framework, *Research progress of extracellular vesicles derived from mesenchymal stem cells in the treatment of neurodegenerative diseases* shifts attention from transplanted cells to mesenchymal stem cell (MSC)-derived extracellular vesicles (EVs). EVs mediate intercellular communication and regulate neuroinflammation. Evidence shows that this advance therapy medicinal product retains key therapeutic properties of their parent cells. These findings suggest that cell-free approaches may overcome limitations of transplantation while preserving beneficial effects. This work broadens the concept of cell therapy by emphasizing signaling mechanisms over cell replacement alone. Moreover, EVs represent one of the most promising examples of next-generation regenerative medicine, highlighting the importance of paracrine communication as a central mechanism through which therapeutic cells influence neural and immune responses.

A similar shift is observed in *Stem cell therapy for stroke: mechanisms, clinical translation, and future perspectives*. In stroke, therapeutic options remain limited beyond acute intervention. This review shows that stem cell therapy is moving from replacement strategies toward modulation of the post-injury environment. Evidence from mesenchymal and neural stem cells supports their role in shaping inflammation and promoting repair. However, poor survival after transplantation remains a challenge. The growing interest in cell-free and bioengineered approaches reinforces the importance of targeting the tissue and immune context. The article further illustrates a broader trend within the field: therapeutic efficacy often derives less from direct cell integration and more from the capacity of transplanted cells to orchestrate endogenous repair pathways through immune and trophic signaling.

This concept is further supported by *Plasticity, injury-induced reprogramming, and translational applications of Schwann cells in neural regeneration*, which adds a glial perspective. Schwann cells display high plasticity and contribute to repair through trophic support, structural remodeling, and interaction with injured tissue, including in the CNS. Their role highlights the importance of glial biology in regeneration. It also emphasizes that effective cell therapy depends on coordinated interactions within the neuroimmune environment, not only on neuronal replacement. By focusing on Schwann cell plasticity and injury-induced reprogramming, this work underscores the growing recognition that glial cells are active participants in tissue regeneration and immune regulation rather than passive support elements.

Together, these contributions converge on a shared principle. Despite differences in strategies, including neural progenitors, EVs, stem cells, and glial-based approaches, their success depends on the context in which they act. Outcomes are shaped by interactions between therapeutic products, resident CNS cells, immune populations, and disease-specific microenvironments. These interactions regulate key processes such as neuroprotection, inflammation, remyelination, and endogenous repair. Notably, the studies collected in this Research Topic consistently point to neuroimmune communication as a central determinant of therapeutic outcome, regardless of the specific cellular platform employed.

In summary, this Research Topic reflects a shift in the field of CNS repair. Cell therapy is no longer viewed only as a replacement strategy. It is better understood as a form of biological communication within diseased neural tissue. Future progress will depend not only on the type of therapeutic product, but also on understanding how it is recognized by the host, how it modulates inflammation, and how it activates endogenous repair pathways in the CNS. Advances in bioengineering, extracellular vesicle technologies, and precision regenerative medicine are likely to further accelerate this transition, enabling increasingly targeted interventions that harness the dynamic interplay between the nervous and immune systems. Ultimately, a deeper understanding of this crosstalk may pave the way for transformative therapies capable of addressing currently unmet clinical needs across a wide range of neurological disorders.

